# New Implications for the Melanocortin System in Alcohol Drinking Behavior in Adolescents: The Glial Dysfunction Hypothesis

**DOI:** 10.3389/fncel.2017.00090

**Published:** 2017-04-05

**Authors:** Juan A. Orellana, Waldo Cerpa, Maria F. Carvajal, José M. Lerma-Cabrera, Eduardo Karahanian, Cesar Osorio-Fuentealba, Rodrigo A. Quintanilla

**Affiliations:** ^1^Centro de Investigación y Estudio del Consumo de Alcohol en AdolescentesSantiago, Chile; ^2^Laboratorio de Neurociencias, Departamento de Neurología, Escuela de Medicina, Facultad de Medicina, Pontificia Universidad Católica de ChileSantiago, Chile; ^3^Laboratorio de Función y Patología Neuronal, Departamento de Biología Celular y Molecular, Facultad de Ciencias Biológicas, Pontificia Universidad Católica de ChileSantiago, Chile; ^4^Unidad de Neurociencia, Centro de Investigación Biomédica, Universidad Autónoma de ChileSantiago, Chile; ^5^Facultad de Kinesiología, Artes y Educación Física, Universidad Metropolitana de Ciencias de la EducaciónSantiago, Chile; ^6^Laboratory of Neurodegenerative Diseases, Universidad Autónoma de ChileSantiago, Chile

**Keywords:** alcohol drinking, melanocortins, neuroinflammation, metabolism and bioenergetics, synaptic dysfunction

## Abstract

Alcohol dependence causes physical, social, and moral harms and currently represents an important public health concern. According to the World Health Organization (WHO), alcoholism is the third leading cause of death worldwide, after tobacco consumption and hypertension. Recent epidemiologic studies have shown a growing trend in alcohol abuse among adolescents, characterized by the consumption of large doses of alcohol over a short time period. Since brain development is an ongoing process during adolescence, short- and long-term brain damage associated with drinking behavior could lead to serious consequences for health and wellbeing. Accumulating evidence indicates that alcohol impairs the function of different components of the melanocortin system, a major player involved in the consolidation of addictive behaviors during adolescence and adulthood. Here, we hypothesize the possible implications of melanocortins and glial cells in the onset and progression of alcohol addiction. In particular, we propose that alcohol-induced decrease in α-MSH levels may trigger a cascade of glial inflammatory pathways that culminate in altered gliotransmission in the ventral tegmental area and nucleus accumbens (NAc). The latter might potentiate dopaminergic drive in the NAc, contributing to increase the vulnerability to alcohol dependence and addiction in the adolescence and adulthood.

## Introduction

Alcohol is the most commonly used and abused drug worldwide (Koob and Le Moal, 2005). According to the Global Information System on Alcohol and Health (World Health Organization, 2014), the annual consumption during 2010 was equal to 6.2 L of pure alcohol per person aged 15 years or older, which implies consumption of 13.5 g of pure alcohol per day. Alcoholism is a complex and multifactorial disorder characterized by a lack of control over excessive alcohol consumption, in spite of its significant negative consequences (Edenberg and Foroud, 2013). Individuals suffering of this disorder exhibit compulsive alcohol use and a loss of behavioral control, as well as alcohol tolerance and withdrawal symptoms, which may include anxiety, depressive episodes, social avoidance, insomnia, nausea and seizures, generating substantial health, societal and economic consequences (Spanagel, 2009). In fact, alcohol abuse causes approximately 3.3 million deaths every year (or 5.9% of all deaths), and 5.1% of the global burden of disease is attributable to this dependence syndrome. Given this, in 2014 the World Health Assembly approved a resolution to urge countries to strengthen their national responses to public health problems caused by the harmful use of alcohol (World Health Organization, 2014).

As occur with other drugs, addiction to alcohol is a chronically relapsing disorder characterized by (i) compulsion to seek and drink alcohol, (ii) loss of control in limiting consume, and (iii) appearance of a negative emotional state (e.g., stress, dysphoria, anxiety) reflecting a motivational withdrawal syndrome when access to alcohol is prevented (defined as dependence) (Koob, 2013; Ron and Barak, 2016). Most of people begin to consume alcohol as part of experimentation and social drinking, which is accompanied of anxiolytic feelings and rewarding, as well as socially facilitating effects (Everitt and Robbins, 2005). When a person repeatedly consumes alcohol, develops tolerance to it and drinking may become more automatic and less voluntary (Everitt and Robbins, 2005). From a neurobiological perspective, in this stage of the addiction cycle, alcohol consumption is a goal-directed behavior, initiated and executed by brain areas within the executive control network, with its rewarding effects processed by appetitive drive regions (Kyzar and Pandey, 2015). These crucial anatomical circuits comprise the mesocorticolimbic dopamine system that originates in the ventral tegmental area (VTA) and projects to the nucleus accumbens (NAc), opioid peptides in the ventral striatum, extended amygdala, and VTA, and glutamate in the dorsolateral prefrontal cortex and anterior cingulate cortex (Koob, 2013). The behavioral transformation between pursuing impulsively alcohol for its rewarding effects (positive reinforcement) and seeking compulsively alcohol in order to remove the negative emotional state associated with withdrawal (negative reinforcement) are clinical features of alcohol addiction (Koob, 2013).

Alcohol abuse not only occurs in the adult population, but is also a well-known health concern during adolescence (Brown and Tapert, 2004). Indeed, information from the WHO’s Global Burden of Disease study reveals that 7.4% of all disabilities and premature deaths in people aged 10–24 years are attributable to alcohol, followed by unsafe sex (4%) or illicit drug use (2%) (Gore et al., 2011). This evidence supports the idea that the onset of drinking at an early age increases the risk of developing an alcohol use disorder in adulthood (DeWit et al., 2000). Currently, several governmental and health institutions worldwide are seeking preventive strategies focused on understanding the etiology of alcohol drinking behavior in young people. Here, we examine the molecular mechanisms that prompt alcohol use and abuse in adolescents by focusing in the possible role of glial cell signaling and melanocortin (MC) system. In particular, we address the signaling pathways that contribute to the switch from moderate to uncontrolled excessive alcohol intake and dependence. As alcoholism is thought to be a maladaptive form of learning and memory that impact whole body homeostasis, we also incorporate possible pathway molecules that have been linked to synaptic plasticity, learning and memory, as well as whole body metabolism.

## Heavy Episodic Drinking in Adolescents and Brain Circuits Involved

In the last years, it has been observed that a growing number of adolescents drink alcoholic beverages with the intention of becoming intoxicated (Center for Disease Control, 2013). This binge drinking practice is characterized by the consumption of large amounts of alcohol over a short time period (minutes to hours), especially during leisure time and at weekends, with periods of abstinence between drinking episodes. In spite of the fact that young people drink less often than adults, most adolescents drink more than twice as much alcohol per drinking episode on average when compared to adults (Center for Disease Control, 2013).

In 2004, the National Institute for Alcohol Abuse and Alcoholism (NIAAA) defined binge drinking as a pattern of alcohol consumption that results in a blood alcohol concentration (BAC) of 0.08 g/dL or greater (NIAAA Newstetter, 2004). Usually people adopting this behavior drink five or more drinks in less than two hours. This heavy drinking pattern produces several short- and long-lasting negative effects in adolescents. According to the Global Burden of Disease Study of 2013, alcohol abuse was the highest risk factor for disability-adjusted life-years (7 % overall, 10.5% for males, and 2.7% for females) for young people aged between 20 and 24 years (Mokdad et al., 2016). In addition, heavy alcohol consumption during adolescence is associated with significant mental health impairment and adverse social effects (McBride and Cheng, 2011), as well as the increased probability of using and abusing other drugs, such as tobacco, marijuana or other illicit drugs (Kirby and Barry, 2012). The relationship between early alcohol consumption in young people and the increased risk of developing alcoholism during adulthood has been well documented. In fact, several studies have reported that alcohol consumption prior to 14 years old produces a 4-fold increase in the risk of becoming alcohol dependent in adulthood (DeWit et al., 2000; Dawson et al., 2008).

Clinical and animal studies have revealed that adolescents are more susceptible to alcohol influence than adults (Donovan, 2004; Spear and Swartzwelder, 2014). Adolescence is the period of life particularly crucial for development of brain circuits responsible for emotion and cognition, involving changes in cortical volume, axonal growth, gene expression, and refinement of cortical connections by a process known as “synaptic pruning” (Tau and Peterson, 2010). The prefrontal cortex and the limbic system are two important networks that exhibit ongoing structural and functional maturation in adolescents and young adults (Arain et al., 2013; Caballero et al., 2016). While the prefrontal cortex is involved in higher cognitive processing related to executive functioning (e.g., planning, goal setting, inhibitory control), decision making, and cognitive-affective behaviors (Gourley and Taylor, 2016), the limbic system govern social and emotional processing and is critical for immediate reward processing (Rolls, 2015). The adolescent brain is particularly susceptible to the detrimental effects of alcohol abuse given the so called “windows of vulnerability” created by the earlier developing limbic system and brain affective regions relative to the later maturation of the prefrontal cortex (Crews et al., 2007). This “maturational lag” produces that when making decisions, adolescents show increased involvement of appetitive/impulsive motivational systems (e.g., drink alcohol for immediate reward), but blunted recruitment of top–down executive controls of the prefrontal cortex (Steinberg, 2007; Crews et al., 2007). The asymmetry between brain areas involved in the impulsive emotionality and those implicated in reflective and executive function may make adolescents more vulnerable to engaging in addictive behaviors, including alcoholism. Interestingly, some studies suggest that most drugs related to addictive behaviors may strengthen this imbalance (Bechara, 2005). This highly sensitivity to positive rewarding effects of alcohol along with the fact that young people are less sensitive to negative aspects of alcohol abuse (e.g., sedative effects); it may explain the excessive alcohol intake during adolescence (Donovan, 2004; Spear and Swartzwelder, 2014). In this context, heavy alcohol drinking during adolescence could exerts long-lasting impacts on adult brain networks, causing different changes including impairment of intellectual function, rational decision making, and emotional maturation.

What brain circuits linked to positive rewarding and appetitive/impulsive function are hyper activated at the initial stages of alcohol intoxication in adolescents? Similar to adults, adolescents that engaged in binge drinking practices exhibit increased dopaminergic function at the VTA and NAc, as well as glutamatergic drive in the prefrontal cortex, which are likely brain regions involved in the reinforcing effects of acute alcohol abuse (Pascual et al., 2009; Maldonado-Devincci et al., 2010; Allen et al., 2011). Another important brain region that undergoes long-lasting neuroadaptive changes during alcohol abuse in adults and adolescents is the hypothalamus (Barson and Leibowitz, 2016). Indeed, different orexigenic neuropeptides acting at the hypothalamus (e.g., galanin, encephalin, orexin) stimulate alcohol consumption by enhancing positive reward and most of them are upregulated by alcohol, which potentiate even further consumption (Rada et al., 2004; Schneider et al., 2007; Barson et al., 2010). Conversely, well-known neuropeptides that have anorexigenic properties, including the endogenous opioid dynorphin, corticotropin-releasing factor, and MCs, inhibit alcohol drinking along with the positive reward achieved by its consumption (Navarro et al., 2003; Thorsell et al., 2005; Barson et al., 2010). The abnormal expression of these neuropeptides and their receptors, besides their impact at the limbic system have emerged as pivotal factors for developing alcohol-related drinking behaviors and thereby, this knowledge has been used also to explain alcohol binge drinking patterns in adolescents (Crews et al., 2000; Slawecki et al., 2004).

Accumulating anatomical, genetic, and pharmacological evidence has shown that the MC pathway in the hypothalamus and other brain areas is critical for developing dependency and addictive behaviors related to alcohol consumption (Olney et al., 2014). However, the molecular and cellular mechanisms behind these changes remain to be fully understood and this is particularly true for the initial stages of alcohol dependency in adolescents.

## Crosstalk Between Alcohol Consumption and Melanocortin System

**FIGURE 1:**
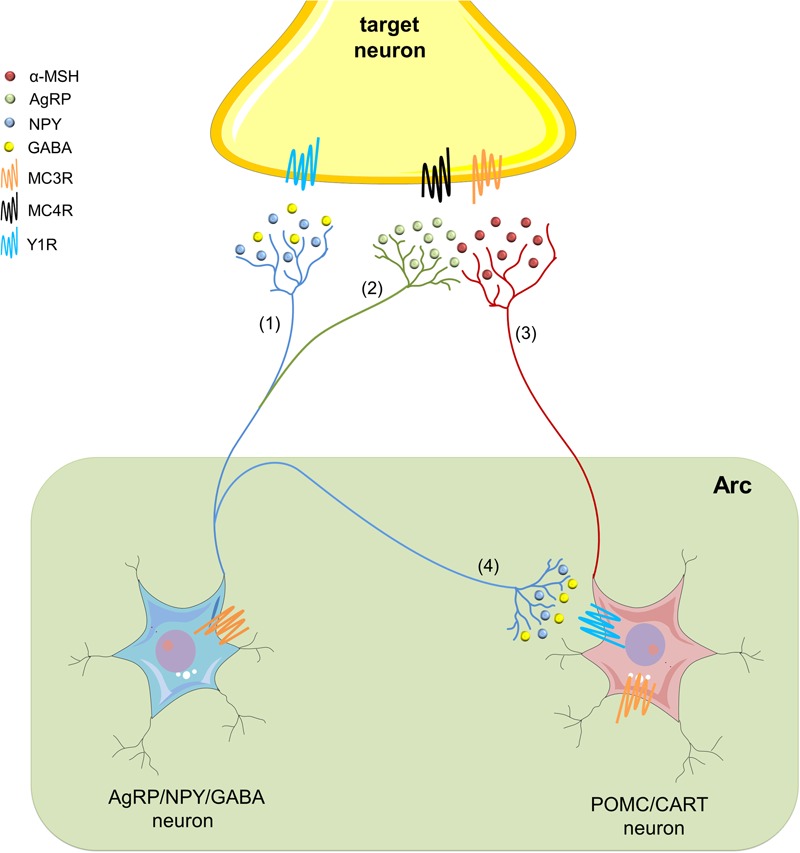
**The hypothalamic melanocortin system.** In the arcuate nucleus of the hypothalamus (Arc), neuropeptide Y/agouti-related protein/γ-amino butyric acid (AgRP/NPY/GABA) neurons (blue) embrace the first-order sensory networks of the melanocortin (MC) system. These neurons project to second-order target areas to regulate multiple physiological functions, including the neurobiological responses to alcohol abuse. NYP (blue) acts on Y1, as well as on Y2 and Y5 receptors (not depicted), whereas via activation of its metabotropic receptors, GABA may establish an inhibitory tone **(1)**. AgRP is a potent endogenous antagonist of MC3Rs and MC4Rs, and therefore, antagonizes actions of α-melanocyte-stimulating hormone (α-MSH) **(2)**. At the other end, pro-opiomelanocortin/cocaine- and amphetamine-regulated transcript (POMC/CART) neurons (red) constitute the other first-order sensory network in the MC system at the Arc. They synthesize α-MSH and release it to activate MC receptors in different second-order regions of the brain **(3)**. In addition, GABA released from NPY/AgRP/GABA neurons suppresses the action of α-MSH by inhibiting POMC/CART neurons at the Arc **(4)**.

Within the arcuate nucleus of the hypothalamus (Arc) and the nucleus of the solitary tract (NST), cleavage of the polypeptide precursor pro-opiomelanocortin (POMC) gives born to different MC peptides (Cone, 2005; Ellacott and Cone, 2006) (**Figure 1**). Among these are α-, β-, and γ-melanocyte stimulating hormones (MSH), as well as adrenocorticotrophic hormone (ACTH) (Hadley and Haskell-Luevano, 1999). In rodents, MC peptides act through at least five receptor subtypes, namely MC1-5R, which are coupled to heterotrimeric G-proteins that stimulate adenylyl cyclase activity (Hadley and Haskell-Luevano, 1999). MC3R and MC4R are the most predominant MCR subtypes expressed in the brain (Mountjoy, 2010b). Immunohistochemical and *in situ* hybridization studies have detected MC4R localization in various brain regions, including the hippocampus, paraventricular nucleus of the hypothalamus (PVN), Arc, ventromedial hypothalamus (VMH), amygdala, VTA, and NAc (Kishi et al., 2003; Liu et al., 2003). Similar evidence has shown the specific expression of MC3Rs in the hypothalamus and the limbic system (Roselli-Rehfuss et al., 1993). Interestingly, both receptors have an endogenous agonist (α-MSH), and an physiological antagonist (agouti-related protein; AgRP) (Haskell-Luevano and Monck, 2001; Nijenhuis et al., 2001; Chai et al., 2003). These transmitters display opposing actions on MC3Rs and MC4Rs, impacting neuronal circuits and further hypothalamic-dependent physiological functions (**Figure 1**).

Multiple studies have revealed the involvement of MC system in the neurobiological response to alcohol consumption. In the first place, α-MSH and other MCs are expressed in different brain areas involved in the neurobiological response to ethanol, including the striatum, NAc, VTA, amygdala, hippocampus, and hypothalamus (Dube et al., 1978; Jacobowitz and O’Donohue, 1978; Bloch et al., 1979; O’Donohue et al., 1979; O’Donohue and Jacobowitz, 1980; Yamazoe et al., 1984). Second, intracerebroventricular infusion of a non-selective MCR agonist, melatonin-II, reduces voluntary ethanol drinking in adult alko alcohol (AA) rats (Ploj et al., 2002) and C57BL/6J mice (Navarro et al., 2003), while the administration of AgRP increases alcohol consumption (Navarro et al., 2003). Importantly, MCR agonists fail in mitigate alcohol intake on mutant mice lacking MC4Rs (Navarro et al., 2011), unveiling the fundamental role of this receptor in dependency and behavioral response to alcohol (Navarro et al., 2011). In agreement with these data, infusion of a selective MC4R agonist (cyclo (NH-CH2-CH2-CO-His-D-Phe-Arg-Trp-Glu)-NH2) at the NAc and VTA, but not into the lateral hypothalamus (LH), diminish voluntary alcohol consumption in rats (Lerma-Cabrera et al., 2012). Follow-up work has demonstrated that MC signaling within the NAc contributes to alcohol consumption by modulating the non-homeostatic aspects (palatability) of intake (Lerma-Cabrera et al., 2013b), which bring to light the key role of MCs in limbic regions implicated in the hedonic response to alcohol.

The interaction between MCs and alcohol is not only limited to the neuromodulatory effect of these transmitters on alcohol consumption, but also implies the well-known regulation of alcohol on MC system. Numerous animal research has demonstrated that ethanol, the predominant alcohol in alcoholic beverages, disturbs the function of MC system depending on how this drug is administered (for review see Olney et al., 2014). For example, acute exposure to ethanol results in a drastic reduction of α-MSH-immunoreactivity in the PVN, Arc, and dorsomedial hypothalamic-dorsal (DMNd) and -ventral (DMNv) nuclei, as well as the central nucleus of amygdala (CeA) (Kokare et al., 2008). Similar findings have been found by others groups in the Arc, LH, CeA, and the paraventricular nucleus of the thalamus (PVT) in rats subjected to acute and chronic treatment with ethanol (Navarro et al., 2008). In addition, chronic exposure to an ethanol-containing diet significantly decreases levels of POMC in the Arc in conjunction with the expression of pro- prohormone convertases 1 (PC1) and 2 (PC2), both responsible for POMC processing (Navarro et al., 2013). In contrast, other works indicate that chronic ethanol treatment significantly raises the α-MSH-immunoreactivity in the PVN, Arc, DMNd, DMNv, and CeA, response that is potentiated following 24 h ethanol withdrawal (Kokare et al., 2008). The reasons for this discrepancy may rely in differences in the dose and duration of ethanol treatment. Another relevant issue that may explain the diverse outcomes regarding the regulation of MC system under different ethanol consumption paradigms is the innate differences. As such, C57BL/6J mice, which display high rates of voluntary ethanol intake, have elevated basal α-MSH immunoreactivity in hypothalamic areas and lower α-MSH expression in the medial amygdala relative to 129/SvJ mice, a strain which exhibit low rates of spontaneous ethanol consumption (Cubero et al., 2010). In the same way, AA rats, which are selectively bred to prefer ethanol, exhibit abnormal expression patterns of MC3Rs in the PVN, Arc, and VMH compared to alko-non-alcohol (ANA) rats (Lindblom et al., 2002). Together these findings suggest that the different patterns of drinking observed amongst these animals may be attributable to innate differences in the function of the MC system.

Nowadays, although plenty of evidence support the involvement of MC system in adult alcoholism, the contribution of this pathway in binge drinking during adolescence is just beginning to be explored (Lerma-Cabrera et al., 2013a). In the following sections, we describe and discuss some of the possible mechanisms underlying this issue.

## Astrocytes and Microglia: Primary Targets of Alcohol Abuse

### Astroglial Dysfunction and Alcohol Abuse

Astrocytes constitute the major glial cell type in the CNS and encompass a far-reaching syncytial network that anatomically and functionally connect neuronal synapses with brain blood vessels (Volterra and Meldolesi, 2005; Barres, 2008; Perea et al., 2009). Astroglial processes, together with pre- and postsynaptic neuronal complexes, embrace the “tripartite synapse” (Schafer et al., 2013). Within this anatomical and functional arrangement, astrocytes sense neuronal activity and respond locally through the release of bioactive molecules termed “gliotransmitters” (e.g., glutamate, ATP, and D-serine) (Perea et al., 2009). Besides to surrounding the synaptic cleft, astrocytes project the well-known specialized terminal processes called “endfeet”, toward multiple vascular elements, including capillaries, intracerebral arterioles and venules (Simard et al., 2003). The above provides to astrocytes with an unparalleled architectural position to favor the local and long distance release of gliotransmitters and vasoactive factors that control different neuronal circuits. Along with their trophic and synaptic role in the brain parenchyma, astrocytes are major protagonists in supplying energy to neurons (e.g., lactate), maintaining the homeostatic balance of extracellular pH, neurotransmitters and ions, as well as controlling the reactive oxygen species (ROS) response and intercellular communication and propagation of Ca^2+^ signaling.

Does alcohol consumption affect astrocyte function? A vast number of studies have shown that astrocytes subjected to *in vitro* and *in vivo* alcohol administration become activated and undergo long-lasting molecular and morphological changes, referred to as reactive astrogliosis (Blanco and Guerri, 2007; Adermark and Bowers, 2016; Saito et al., 2016). This phenomenon constitutes a graded, multistage and evolutionarily conserved astroglial reaction that counteract acute damage, restoring the homeostasis and limiting the brain parenchyma injury (Pekny and Nilsson, 2005). Along with hypertrophy of astrocytes processes and enlargement of the intermediate filament network via upregulation of glial fibrillary acidic protein (GFAP), this reaction also involves disturbances on astroglial functions such as altered gliotransmission and Ca^2+^ signaling, elevated production of cytokines and nitric oxide (NO) (Pekny and Nilsson, 2005). Despite that reactive astrogliosis is an adaptive mechanism of protection, when it persists, can turn into a detrimental response, leading to neuronal damage and recruitment of the innate immune response.

In terms of astrocyte number and GFAP expression, ethanol seems to induce different outcomes depending on developmental period, addiction stage, brain region and method of ethanol administration (e.g., amount and periodicity of exposure) (Bull et al., 2015). For example, adult rats exposed to repeated gavage of ethanol or ethanol-containing diet exhibit an increased number of GFAP positive astrocytes in the cerebral cortex (Dalcik et al., 2009; Udomuksorn et al., 2011) and similar findings have been observed at the prelimbic and anterior cingulate cortex (Bull et al., 2014) or during ethanol abstinence at the prelimbic cortex (Miguel-Hidalgo et al., 2006) and Nac (Bull et al., 2014). In contrast, prelimbic and orbitofrontal prefrontal cortex of rats that had continuous access to ethanol show a reduction in GFAP positive astrocytes after 3-week abstinence, whereas astrocyte density decreases at the anterior cingulate and orbitofrontal cortex during abstinence in a model of operant ethanol self-administration (Bull et al., 2015). In the same manner, a diminished astrocyte number is also found in the rat dorsolateral and orbitofrontal prefrontal cortex (Miguel-Hidalgo et al., 2002, 2006) and hippocampus of human alcoholics (Korbo, 1999). These changes also take place in adolescents and young adults. Indeed, ethanol exposure increases the expression of GFAP and the number of astrocytes in the hippocampus, corpus stratum and frontal cortex of adolescent rodents (Evrard et al., 2006; Kane et al., 2014), whereas a recent study has found that chronic ethanol administration during adolescence reduced GFAP positive astrocytes at the CA3 hippocampal area and hilus sub-regions (Oliveira et al., 2015). As mentioned before, these conflicting results may be linked to differences in time of ethanol exposure, amounts and methods of administration.

Treatments with alcohol cause profound alterations in cell-to-cell coupling and electrophysiological properties of astrocytes. In fact, ethanol inhibits gap junctional coupling among astrocytes, whereas also blunts their slope of conductance, increase their input resistance and decreased their capacitance without affecting the resting membrane potential (Adermark et al., 2004; Adermark and Lovinger, 2006). In addition, acute ethanol exposure induces astroglial swelling and intracellular free Ca^2+^ concentration ([Ca^2+^]_i_) transients (Allansson et al., 2001), whereas Gonzalez and colleagues linked this response with ROS production and further increased expression of GFAP (Gonzalez et al., 2007). Follow-up studies revealed that ethanol-induced [Ca^2+^]_i_ oscillations triggers the release of glutamate in astrocytes (Salazar et al., 2008), but inverse effects have been seen when they are pre-incubated with ethanol and further kainate-dependent release of glutamate is analyzed (Santofimia-Castaño et al., 2011). At one end, this ethanol-mediated regulation of extracellular glutamate has been attributed to alterations in the expression and function of astroglial excitatory amino acid transporters GLAST and GLT-1 in brain regions linked to positive reward, including the Nac (Smith and Zsigo, 1996; Zink et al., 2004; Alhaddad et al., 2014; Smith et al., 2014; Das et al., 2015; Sari et al., 2016). These transporters remove glutamate from the extracellular environment and thereby, their function is crucial because excessive glutamate can lead to synaptic dysfunction and neuronal excitotoxicity (Ayers-Ringler et al., 2016). Ethanol blunts adenosine uptake via the inhibition of astrocytic nucleoside transporter 1 (ENT1), leading to increased levels of adenosine and activation of purinergic receptors, which result in the downregulation of GLT-1 and further enhanced levels of extracellular glutamate (Wu et al., 2010, 2011; Nam et al., 2012). At the other end, disturbances in extracellular levels of glutamate and other gliotransmitters have been associated to ethanol-mediated astroglial swelling. Indeed, acute treatment with ethanol triggers astrocyte swelling via Na^+^/K^+^/2Cl^-^ cotransporter or the Na^+^/K^+^-ATPase, resulting in the release of glutamate, aspartate and taurine (Kimelberg et al., 1993; Allansson et al., 2001; Aschner et al., 2001a,b; Vargova and Sykova, 2014). Relevant to this point, extracellular levels of taurine are crucial for the ethanol-induced dopamine release in the Nac (Ericson et al., 2011). Furthermore, positive reward caused by impulsive alcohol consumption are associated with aquaporin-4 (AQP4) function (Lee et al., 2013) and its expression correlates with dopamine levels in the Nac (Kuppers et al., 2008), and suppression of cell swelling mitigates ethanol-induced dopamine release (Adermark et al., 2011). Altogether this evidence suggests that gliotransmitter release associated to transporters or astrocyte swelling is modulated by ethanol and could be determinant in regulating synaptic transmission in brain areas related to alcohol consumption.

A number of studies by Guerri’s group and others, have shown that *in vitro* or *in vivo* treatment with ethanol augments the function and/or expression of different inflammatory mediators in astrocytes, including cyclooxygenase 2 (COX_2_), cytochrome P4502E1, inducible NO synthase (iNOS), NO, IL-1β, and TNF-α (Montoliu et al., 1995; Blanco et al., 2004; Valles et al., 2004). Importantly, these effects base on the activation of different cellular pathways including the nuclear factor κB (NF-κB), IL-1β receptor type I (IL-1RI) and toll-like receptor type 4 (TLR4) (Blanco et al., 2004, 2005, 2008; Alfonso-Loeches et al., 2010). In particular, the ethanol-induced upregulation of iNOS, COX_2_, and IL-1β occurs via the stimulation of RhoE, as well as IRAK and MAP kinases, such as ERK1/2, p-38, and JNK, which trigger the downstream activation of oxidant-sensitive transcription factors NF-κB and AP-1 (Valles et al., 2004; Guasch et al., 2007). Alterations in the expression of pro-inflammatory immune genes occur in postmortem brain from alcoholics and animals exposed to alcohol, whereas molecules known to reduce inflammation have shown to ameliorate alcohol-mediated behaviors in animal models (Mayfield et al., 2013; Cui et al., 2014; Truitt et al., 2016; Nennig and Schank, 2017). Increased free radical production and low antioxidant levels are major features of alcohol-induced brain damage (Crews et al., 2000). At the CNS, mechanisms of antioxidant defense and metabolic homeostatic balance largely depend on glial cells, in particular astrocytes (Fernandez-Fernandez et al., 2012). Astrocyte-to-astrocyte signaling protects neurons against oxidative injury by suppressing the accumulation of free radicals and stabilizing Ca^2+^ homeostasis in neurons (Blanc et al., 1998). During early stages of alcohol consumption, astrocytes may help to compensate the alcohol abuse-induced disturbances in redox balance and antioxidant mechanisms. Accordingly, they prevent ethanol-induced neuronal death by maintaining glutathione (GSH) homeostasis (Watts et al., 2005; Narasimhan et al., 2012). Nonetheless, in situations of chronic and progressive alcohol abuse, ethanol may impair astroglial function, thus altering antioxidant and metabolic coupling between neurons and astrocytes. Supporting this line of thought, ethanol acutely reduces astrocytic gap junction coupling (Adermark et al., 2004; Adermark and Lovinger, 2006), and induces the production of free radicals, and further oxidative stress in astrocytes (Montoliu et al., 1995; Russo et al., 2001; Muscoli et al., 2002; Salazar et al., 2008). Under this view, anomalies in the inflammatory and antioxidant profile of astrocytes, along with the impairment of immune function of microglia (see next section), may be vital for the function of brain networks involved in alcohol reward and dependency.

### Microglia-Mediated Inflammation and Redox Imbalance during Alcohol Abuse

Microglia comprises almost 5–15% of the entire number of brain cells and are the predominant pieces of the innate immune system at the CNS (Lawson et al., 1990). Originating from mielomonocytic precursor cells of the hemangioblastic mesoderm, microglia populates the brain parenchyma prior to the developmental closure of the blood–brain barrier (BBB) (Ginhoux et al., 2010). In the normal brain, most microglia displays a “resting” surveillance nature, which correlates with a dynamic environmental pursuing and unceasing seeking of exogenous or endogenous signals constituting a brain threat (Streit, 2001; Kettenmann et al., 2011). When homeostatic equilibrium is disturbed, the resting features of microglia turn into a reactive phenotype implicating a wide array of modifications in different microglial functions, including proliferation, morphology, motility, migration, proteostasis, phagocytosis and intercellular communication (Hanisch, 2002; Block et al., 2007). This complex number of changes is denominated as “microglial activation” and embrace large-scale and functional remodeling that depend on the nature, intensity and duration of the stimulus (Ransohoff and El Khoury, 2015). At this point, microglia becomes an unrestrained core of inflammatory mediators (e.g., cytokines and free radicals) that drive neuronal damage rather than exhibiting a repair-orientated profile (Block et al., 2007). Although an efficient immune response is necessary to resolve brain threats, under these circumstances, dysfunctional microglia can induce detrimental processes leading to the subsequent recruitment of other cell types involved in the innate immune response. This may worsen disease progression by altering synaptic function, ion homeostasis, antioxidant defense and cell survival (Block et al., 2007).

As mentioned before, a broad number of data have shown that alcohol elevates inflammation in the brain, contributing to the impaired neurological function and neurodegeneration associated with alcohol consumption (Yakovleva et al., 2011). From this angle and given their inflammatory properties, microglia arise as crucial players in the onset and progression of alcohol-induced neuronal dysfunction and behavioral abnormalities (Chastain and Sarkar, 2014; Yang et al., 2014; Suk, 2007). Considerable evidence has described that ethanol triggers microglial activation in cell cultures (Fernandez-Lizarbe et al., 2009; Alfonso-Loeches et al., 2010; Boyadjieva and Sarkar, 2010, 2013; Fernandez-Lizarbe et al., 2013; Alfonso-Loeches et al., 2016), animal models (Alfonso-Loeches and Guerri, 2011; McClain et al., 2011; Qin and Crews, 2012a; Zhao et al., 2013; Ahlers et al., 2015; Alfonso-Loeches et al., 2016) and postmortem brains of alcoholics (He and Crews, 2008; Byun et al., 2014; Coleman et al., 2017). Indeed, ethanol increases the number of microglia showing large cell bodies and thick processes characteristic of activated morphology (Nixon et al., 2008; Fernandez-Lizarbe et al., 2009; McClain et al., 2011; Ahlers et al., 2015) and most of these changes are accompanied with elevated expression of pro-inflammatory cytokines, including TNF-α, MCP-1, and IL-1β, as well as neuronal damage (Alfonso-Loeches and Guerri, 2011; Boyadjieva and Sarkar, 2013; Lippai et al., 2013a; Zhao et al., 2013; Byun et al., 2014; Coleman et al., 2017).

A crucial role for microglia-mediated inflammation has been attributed to TLR activation, cytokine production and NF-κB signaling. In particular, TLR4/TLR2 are required for ethanol-induced activation of microglia and subsequent release of IL-1β, TNF-α, MIP-1α, MIP-2, and IL-6 and other inflammatory mediators (NO and free radicals), which in turn, lead to neuronal apoptosis (Fernandez-Lizarbe et al., 2009, 2013; Alfonso-Loeches et al., 2010; Boyadjieva and Sarkar, 2010). Essential for these processes is the upregulation of NF-κB and recruitment of TLR4/TLR2 into the lipid rafts, along with the stimulation of p38 MAP kinase, IRF-3, STAT-1/IRF-1, iNOS, and COX_2_ pathways (Fernandez-Lizarbe et al., 2009, 2013). Furthermore, *in vivo* and *in vitro* ethanol exposure fails to trigger neuroinflammation, microglial activation, myelin alterations and neural death in TLR4 knockout cultures and mice (Fernandez-Lizarbe et al., 2009; Alfonso-Loeches et al., 2010, 2012). On the other hand, although some findings indicate that chronic ethanol exposure does not alter microglial proliferation (Dlugos and Pentney, 2001; Riikonen et al., 2002; Valles et al., 2004), other studies have shown the opposite during alcohol withdrawal and different periods of abstinence (Nixon et al., 2008; Saito et al., 2010; McClain et al., 2011; Zhao et al., 2013; Alfonso-Loeches et al., 2016).

Microglia are a major source of ROS and free radicals at the CNS. Excessive production of these mediators is a major hallmark of postmortem brain tissue from alcoholic people and likely one the major causes of neuroinflammation and activation of signaling cascades that lead to cell damage and further apoptosis (Upadhya et al., 2000; Matsumoto and Matsumoto, 2008). In fact, uncontrolled consumption of alcohol leads to redox imbalance, encompassed by a high production of oxidants and low levels of antioxidants, and functional alterations in several antioxidant enzymes and molecules, including glutathione (GSH), glutathione peroxidase (GSH-Px), superoxide dismutase (SOD), and catalase (Heaton et al., 2003; Boyadjieva and Sarkar, 2013). Changes in these molecules directly correlate with mitochondrial dysfunction and further neuronal damage (Heaton et al., 2003; Boyadjieva and Sarkar, 2013). Following exposure to ethanol, microglia show increased activity of NADPH oxidase (NOX) (Block, 2008). This enzyme regulates the production of ROS in microglia, with potentially significant consequences for neuronal survival (Block, 2008; Choi et al., 2012). Alcohol consumption increases the production of ROS in activated microglia and astrocytes, resulting in impaired neuronal function and cell death (Qin and Crews, 2012b). Complementary studies from Boyadjieva and Sarkar (2013) showed that concentrations of ethanol ≥25 mM induce neuronal apoptosis via microglia and a mechanism involving oxidative stress, since treatment with antioxidant agents (e.g., GSH, catalase, and SOD) successfully suppressed these effects (Boyadjieva and Sarkar, 2013). Given the complex physiology of microglia, beyond doubt, the impact that alcohol may cause on the inflammatory and redox properties of these glial cells will rely on how it is administrated, its concentration and the timeline in where the observations are made.

## Neuroinflammation, Oxidative Stress, and Glia-To-Neuron Miscommunication: Implications of Melanocortin System in Alcohol Abuse in Adolescents

### Anti-inflammatory Action of Melanocortins and Their Impact on Glial Cells

A substantial body of work has established *in vivo* and *in vitro* the anti-inflammatory features of MCs in different systemic and neuroinflammatory models (Catania et al., 2004, 2010; Catania, 2008). Indeed, α-MSH diminishes fever and inflammation in models of acute, chronic, and systemic inflammation (Lipton et al., 1999), whereas similar protective findings have been observed for different brain pathologies, including Alzheimer’s disease (AD) (Giuliani et al., 2014), traumatic brain injury (Schaible et al., 2013), experimental autoimmune encephalomyelitis (Mykicki et al., 2016), and cerebral ischemia (Giuliani et al., 2006). Furthermore, systemic administration of α-MSH abrogates brain inflammation and cytokine expression evoked by cerebral ischemia or LPS (Rajora et al., 1997; Huang and Tatro, 2002), much as α-, β-, and γ-MSH reduced the production of NO, PGE_2_ during different inflammatory conditions (Weidenfeld et al., 1995; Muceniece et al., 2004; Cragnolini et al., 2006). Albeit MC3R and MC4R expression at the CNS is predominant, until now, diverse lines of evidence indicate that protective effects of MCs depend on the activation of the latter receptor. Pioneering studies by Caruso and colleagues revealed that central administration of α-MSH prevent the LPS-mediated induction of iNOS and COX_2_ gene expression at the hypothalamic level, an effect that occurred via the activation of MC4Rs (Caruso et al., 2004). Similarly, agonists of MC4Rs counteract neuroinflammation and cell damage (Giuliani et al., 2006, 2014, 2017; Spaccapelo et al., 2011; Liu et al., 2015), whereas its pharmacological blockade or downregulation prevent the neuroprotective effects of α-MSH or its analogs and worse the outcome in different brain disease models (Giuliani et al., 2006; Zhang et al., 2015). While the neuroprotective actions of α-MSH/MC4R pathway are not completely understood, it has been proposed that they are in part exerted by inhibiting the production of inflammatory mediators from glial cells (Caruso et al., 2007). Supporting this idea, both astrocytes and oligodendrocytes exhibit important levels of MC4Rs (Caruso et al., 2007; Selkirk et al., 2007; Benjamins et al., 2013), whereas microglia express all isoforms of MC receptors (Delgado et al., 1998; Lindberg et al., 2005; Benjamins et al., 2013).

In astrocytes, α-MSH increases the production of cAMP and proliferation, as well as morphological features that resemble differentiation (Evans et al., 1984; Zohar and Salomon, 1992). In addition, selective activation of MC4Rs suppress the production of NO and PGE_2_, as well as the apoptosis triggered by LPS and IFN-γ in astrocytes (Caruso et al., 2007). In the same line, MC-dependent MCR4 stimulation blunts astroglial activation (Aronsson et al., 2006, 2007; Niu) and enhance the expression of BDNF in these glial cells through a cAMP-PKA pathway (Caruso et al., 2012). In the case of microglia, α-MSH inhibits the release of TNF-α, IL-6, and NO (Delgado et al., 1998; Galimberti et al., 1999), while its analogs stimulate the production of the anti-inflammatory cytokines IL-10 and TGF-β from microglia and astrocytes (Carniglia et al., 2013). Recently, Giuliani and coworkers demonstrated that stimulation of MC4Rs prevents the neurodegenerative changes seen in the triple-transgenic (3xTg-AD) mice, an animal model of AD (Giuliani et al., 2014). These responses were associated to decreasing levels of oxidative and nitrosative species, as along with reduction in the phosphorylation of tau protein and modulation of the inflammatory and apoptotic cascades that are implicated in AD (Giuliani et al., 2014).

As mentioned in previous sections, alcohol consumption strongly reduces the expression of α-MSH in the limbic system and hypothalamus (Olney et al., 2014), whereas MC4R activation within the Nac suppress ethanol drinking (Navarro et al., 2011; Lerma-Cabrera et al., 2013b). In spite of this evidence, it is unknown whether alcohol addiction, in particular during the adolescence, occurs due to an imbalance in the inflammatory profile of glial cells caused by low signaling of the MC system. In the next section, we propose a possible mechanism by which lower drive of MC system may increase inflammatory and activated status of glial cells, resulting in impaired glia-to-neuron communication.

### Decreased Drive of Melanocortin System and Its Effect on Pro-inflammatory Profile of Glial Cells and Gliotransmission

A new line of evidence suggests that endogenous α-MSH may exert an inhibitory tone on different inflammatory mediators via MC4Rs, acting as a local anti-inflammatory agent within the hypothalamus (Caruso et al., 2004). Supporting this idea, multiple neuroprotective actions of MCs and their analogs reside in the suppression of canonical inflammatory pathways in glial cells such as NF-κB, iNOS, and COX_2_, a phenomenon that fail when blockade or downregulation of MCRs occurs (Delgado et al., 1998; Galimberti et al., 1999; Caruso et al., 2004, 2007; Giuliani et al., 2006; Spaccapelo et al., 2011). Given that *in vitro* or *in vivo* treatment with ethanol augments the inflammatory profile of glial cells, it is plausible speculate that this phenomenon may arise as reflex of decreasing anti-inflammatory drive of the MC system. Indeed, although downregulation of MCRs during ethanol consumption has not been yet truly demonstrated, a substantial body of evidence indicates that ethanol administration reduces the expression of α-MSH in the Arc, CeA, PVT, and LH (Rainero et al., 1990; Kokare et al., 2008; Navarro et al., 2008). Whether reduced α-MSH expression triggered by ethanol could be critical for developing early stages of alcohol addiction and whether this take place due the lacking inhibitory tone of MCs on the inflammatory profile of glial cells remain unknown.

There are some clues that strengthen the potential role of neuroinflammation in the onset and progression of alcohol addiction. For instance, pro-inflammatory molecules, including cytokines and chemokines, reinforce alcohol drinking (Blednov et al., 2012), whereas the opposite is observed when anti-inflammatory molecules are administrated (Blednov et al., 2011). Together these findings argue that pro-inflammatory mediators trigger persistent alcohol intake, which may in turn be the result of deficient α-MSH signaling and subsequent glial inflammation. In line with this, minocycline, a well-known inhibitor of microglial activation and inflammatory mediators, reduces ethanol drinking (Agrawal et al., 2011), while at Nac, astrocytes increase [Ca^2+^]_I_, modulating the motivation to self-administer ethanol (Bull et al., 2014). How the decreased drive of MC system is connected to glial inflammation and further reinforcement of alcohol consumption? We believe that downregulation of α-MSH signaling may disturb the inflammatory profile and function of glial cells, resulting in further impaired communication with neurons located in brains areas that are crucial for alcohol rewarding and are more susceptible during adolescence. Different studies have shown that inflammatory mediators disturb intracellular Ca^2+^ dynamics in glial cells, thus affecting the release of gliotransmitters and further impairing the crosstalk between neurons and glial cells (Ida et al., 2008; Wuchert et al., 2009). Taking into account that glial cells are persistently activated in animal models of alcohol consumption (Yakovleva et al., 2011), it is possible that impairment of intracellular pathways and coordination between glial cells and neurons could play an essential role in brain dysfunction observed in alcohol use disorders.

Recent studies have reviewed the potential impact of astrocytes and microglia in the onset and progression of alcohol disorders (Yang et al., 2014; Adermark and Bowers, 2016). Here we do not overview all of this evidence discussed elsewhere, but rather focus on a particular mechanism of gliotransmission that is known to be altered during inflammatory conditions: the hemichannel-mediated paracrine signaling. Hemichannels are plasma membrane channels constituted by a six-fold ring of connexin monomers and serve as aqueous pores permeable to ions and small molecules, providing a diffusional pathway of exchange between intra- and extracellular compartments (Montero and Orellana, 2015). Connexins are abundantly expressed in brain cells and belong to a highly conserved protein family encoded by 21 genes in humans and 20 in mice, with orthologues in other vertebrate species (Abascal and Zardoya, 2013). In the last decade, another gene family encoding a set of three membrane proteins termed pannexins was identified (Bruzzone et al., 2003). Although connexins and pannexins do not share significant amino acid sequences, they have similar secondary and tertiary structures and most of evidence indicates that pannexins form single membrane channels, similar to connexin hemichannels (Sosinsky et al., 2011). In the normal brain, hemichannels and pannexons mediate the physiological release of gliotransmitters (e.g., ATP, glutamate, D-serine, lactate), serving as crucial players during ischemic tolerance, fear memory consolidation, synaptic transmission, neuronal oscillations and glucose sensing (Cheung et al., 2014). Nevertheless, the uncontrolled opening of these channels seem to be critical to the initiation and maintenance of the homeostatic imbalances that are observed in diverse CNS diseases (Salameh et al., 2013; Orellana et al., 2014a, 2016).

**FIGURE 2:**
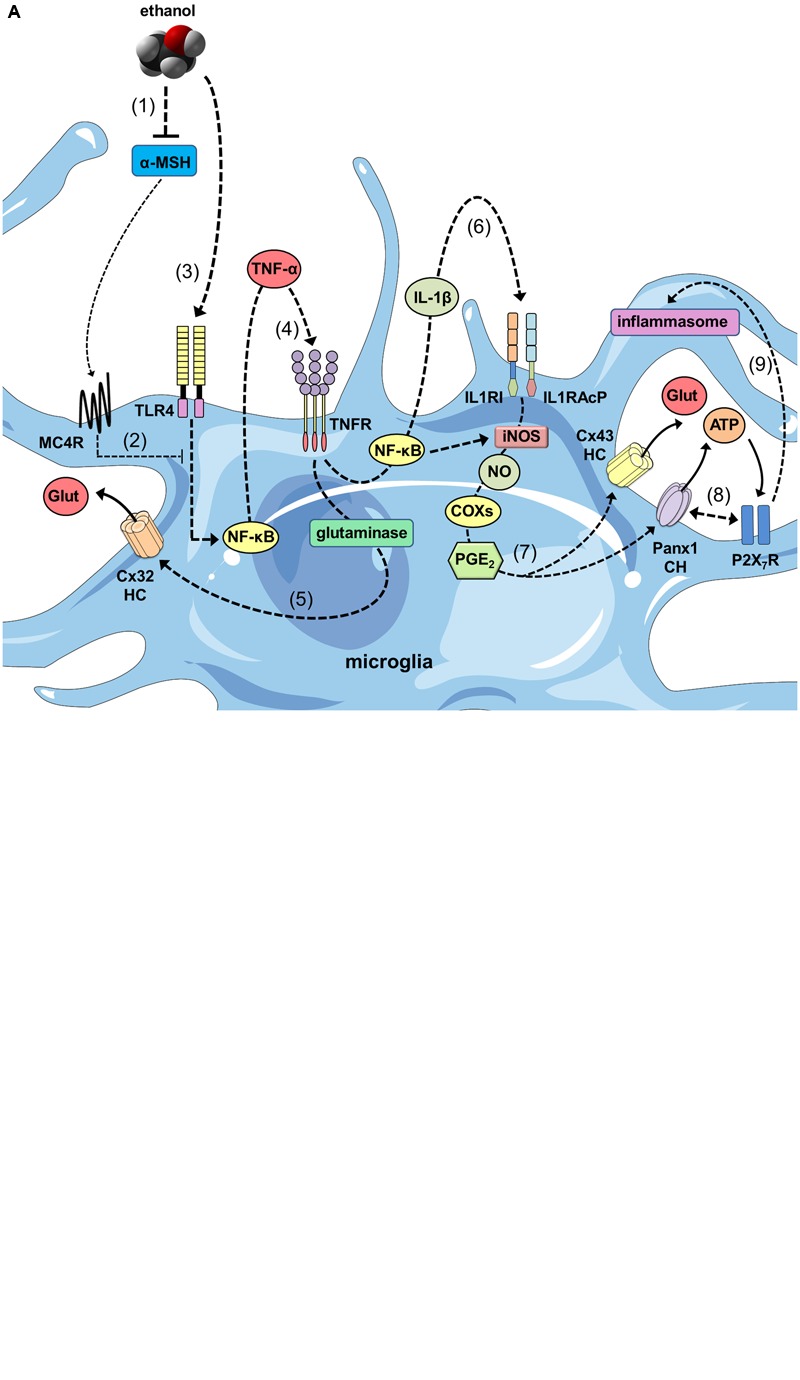
**The ethanol-induced decrease in α-MSH drive and its impact on glial inflammation and hemichannel/pannexon-dependent gliotransmission. (A)** At one end, ethanol reduces the brain levels of α-MSH (1), decreasing the MC4R-mediated anti-inflammatory drive of melanocortin (MC) system on microglia (2). In parallel, ethanol stimulates TLR4Rs (3), resulting in the activation of NF-κβ pathway and further autocrine/paracrine release of TNF-α, *(which acts upon its receptor TNFR1 (4). The latter leads to the activation of glutaminase and the consequent release of glutamate through Cx32 hemichannels (HCs) (5). Similarly, NF-κβ signaling promotes the autocrine/paracrine release of IL-1β, which stimulates its receptor as well as accessory proteins (IL1RI and IL1RAcP) (6), resulting in iNOS activation, NO production, COX activation and PGE_2_ production via unknown mechanisms. PGE_2_ released by microglia binds to the EP1 metabotropic receptor (not depicted) to elicit Ca^2+^ release from intracellular stores (7). This release increases [Ca^2+^]_i_, which is known to open Cx43 HCs and Panx1 channels (CHs) and subsequently the release glutamate and ATP through them. Furthermore, protein-to-protein interactions between Panx1 CHs and P2X_7_Rs trigger the signaling that activate the inflammasome (9), perpetuating the cycle of maturation and secretion of pro-inflammatory mediators (e.g., IL-1β), as well as the uncontrolled release of gliotransmitters during ethanol consumption. **(B)** As with microglia, decreased levels of α-MSH caused by ethanol (1), blunt the MC4R-mediated anti-inflammatory drive of MC system on astrocytes (2). At the same time, ethanol activates TLR4Rs (3) and NF-κβ pathways, establishing the interrelated autocrine/paracrine release of TNF-α (4) and IL-1β (5), similar to what described for microglia. The latter results in the activation of p38 MAP kinase and NO production, as well as the opening of Cx43 hemichannels via s-nitrosylation of Cx43 and release of taurine and glutamate (6). Increases in [Ca^2+^]_i_, which is known to open Panx1 CHs may also evoke the release glutamate through them. In addition, pro-inflammatory cytokines released from microglia could potentiate the activation of these pathways, perpetuating the dysfunctional release of gliotransmitters during ethanol consumption (7).*)

How hemichannels/pannexons could be involved in the miscommunication of glial cell and neurons during ethanol disorders? We speculate that reduced levels of α-MSH caused by ethanol may unlock the tonic inhibition of this neuropeptide on NF-κB pathways, particularly, in glial cells (**Figure 2**). In this context, the well-known stimulant effect of ethanol in the generation of inflammatory mediators (e.g., cytokines and ROS) could alter the functional state of glial hemichannels and pannexons. According with this line of though, different independent groups have shown that NF-κB-mediated pro-inflammatory mediators promote the opening of hemichannels and pannexons in glial cells. Pioneering observations by Takeuchi et al. (2006) described that TNF-α elicits the release of glutamate via Cx32 hemichannels in microglia, resulting in neuritic beading and neuronal death, while comparable results have been found in human microglial CHME-5 cells (Shaikh et al., 2012). Similarly, TNF-α plus IFN-γ increment the expression of Cx43 and Panx1 in EOC20 microglial cells in conjunction with the activation of hemichannels and pannexons (Sáez et al., 2013). Furthermore, the mixture of TNF-α and IL-1β increases the opening of astroglial Cx43 hemichannels by a mechanism depending on the activation of p38 MAP kinase pathway and further production of NO (Retamal et al., 2007; Abudara et al., 2015). With this in mind, it is reasonable to theorize that glial activation elicited by ethanol may induce the opening of hemichannels and pannexons via autocrine release of cytokines and further stimulation of diverse downstream inflammatory mediators such as NO, prostaglandins, ATP, and ROS. Relevant to this point, increased levels of [Ca^2+^]_i_, iNOS, and COX_2_ activation, as well as production of NO, underpin the Panx1 channel-dependent release of ATP in LPS-stimulated microglia (Orellana et al., 2013), whereas NO-mediated Cx43 s-nitrosylation is pivotal in the activation of astroglial hemichannels triggered by oxidative stress (Retamal et al., 2006). Importantly, the stimulation of these pathways has been linked to glial hemichannel/pannexon activation under different pathological conditions, including amyloid β treatment (Gajardo-Gómez et al., 2017), prenatal inflammation (Avendaño et al., 2015), restraint stress (Orellana et al., 2015), spinal cord injury (Garré et al., 2016), high cholesterol diet (Orellana et al., 2014b), AD (Yi et al., 2016), and Niemann-Pick type C disease (Sáez et al., 2013).

Because the above inflammatory mediators (cytokines, ROS, NO, ATP) are elevated during alcohol drinking (Fernandez-Lizarbe et al., 2009, 2013; Alfonso-Loeches et al., 2010; Boyadjieva and Sarkar, 2010), their role may be critical for the possible deregulation of hemichannel/pannexon-mediated gliotransmission (**Figure 2**). An important aspect is the modulatory action that microglia exert on astroglial hemichannel/pannexon activity, which seems to be decisive for neuronal function and survival (Montero and Orellana, 2015; Gajardo-Gómez et al., 2016; Orellana et al., 2016). In fact, microglia subjected to inflammatory conditions release TNF-α and IL-1β, resulting in the further increase of Cx43 hemichannel currents in astrocytes in cell cultures and hippocampal slices (Retamal et al., 2007; Abudara et al., 2015). Interestingly, microglia-evoked Cx43 hemichannel opening allow Ca^2+^ entry and further release of glutamate, affecting excitatory synaptic activity in the hippocampus (Abudara et al., 2015). In the same manner, the release of ATP via astroglial Cx43 hemichannels and/or Panx1 channels (Braet et al., 2003; Iglesias et al., 2009; Garré et al., 2010) comprises a fundamental signaling through which astrocytes control microglial behavior (Verderio and Matteoli, 2001; Schipke et al., 2002). Acting on P2X_7_Rs, ATP evokes Ca^2+^-dependent ATP release in microglia, as acute application of this gliotransmitter induces the opening of Cx43 hemichannels and Panx1 channels in these cells (Bernier et al., 2012; Sáez et al., 2013). Despite of P2X_7_Rs increase [Ca^2+^]_i_ (Baroja-Mazo et al., 2013); a well-accepted condition that opens Cx43 hemichannels and Panx1 channels (Locovei et al., 2006; De Bock et al., 2012); the ATP-induced release of ATP linked to Panx1 channel opening imply protein-protein interactions between this pannexon and P2X_7_Rs (Locovei et al., 2007). Noteworthy, P2X_7_R-dependent opening of Panx1 channels has been related to the secretion of IL-1β by a mechanism engaging the activation of the inflammasome (Pelegrin and Surprenant, 2006; Kanneganti et al., 2007). Indeed, in neurons and astrocytes, opening of Panx1 channels triggers caspase-1 activation in association with components of the multiprotein inflammasome complex, including the P2X_7_R (Silverman et al., 2009; Murphy et al., 2012; Minkiewicz et al., 2013). Remarkably, both purinergic receptors and the inflamasome have been shown to be activated by ethanol *in vitro* and *in vivo* in glial cells and neurons (Lippai et al., 2013b; Alfonso-Loeches et al., 2014; Wang et al., 2015).

Recent studies have revealed that gliotransmission through hemichannels and pannexons is crucial for synaptic transmission and consolidation of fear and spatial memory (Prochnow et al., 2012; Ardiles et al., 2014; Chever et al., 2014; Walrave et al., 2016). Nevertheless, over activation of these channels has been associated to the release of large amounts of gliotransmitters (e.g., glutamate and ATP), resulting in neuronal dysfunction and even with excitotoxicity. We hypothesize that uncontrolled opening of glial hemichannels and pannexons may be a relevant downstream target that disturbs proper glia-to-neuron communication, affecting synaptic transmission in neural circuits crucial for alcohol rewarding.

### Impaired Gliotransmission Mediated by Hemichannels and Pannexons and Its Impact on Neural Circuits Linked to Alcohol Reward in Adolescents

**FIGURE 3:**
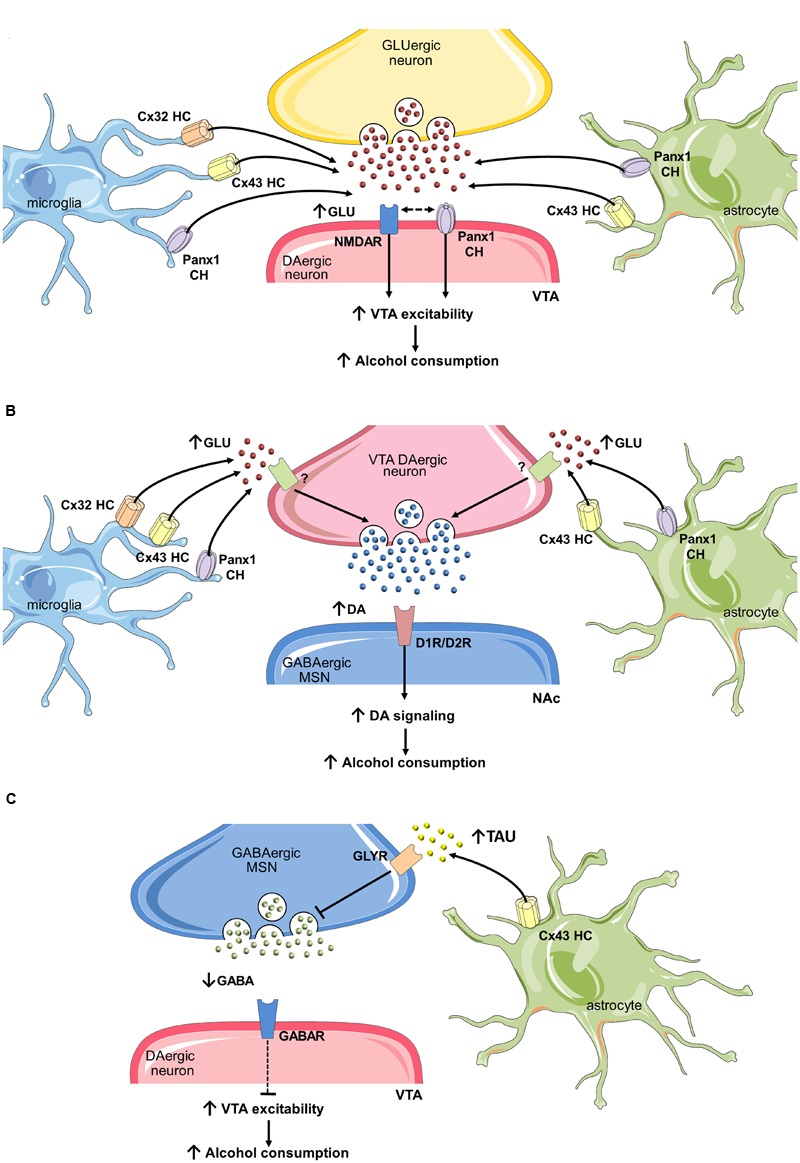
**Uncontrolled opening of hemichannels/pannexons and their effect on neuronal circuits that govern ethanol consumption. (A)** One mechanism by which glial hemichannels or pannexons may increase ventral tegmental area (VTA) dopaminergic neuron activity and nucleus accumbens (NAc) dopamine (DA) levels involves the synaptic release of glutamate via these channels. Stimulation of NMDARs could then augment VTA dopaminergic activity and trigger the firing of GABAergic neurons in the NAc, increasing alcohol consumption. **(B)** In addition, increased glutamate released from glial hemichannels and pannexons may enhance DA levels at the NAc by activation of unknown glutamate receptors on presynaptic dopaminergic terminals at the VTA. Potentiated activation of dopaminergic D1or D2 receptors (D1R/D2R) on medium spiny neurons (MSNs) at the NAc may then promote alcohol consumption. **(C)** Finally, taurine release through astroglial Cx43 hemichannels may activate glycine receptors on MSNs GABAergic neurons, decreasing their inhibitory tone on VTA dopaminergic neurons.

As mentioned in previous sections, the mesocorticolimbic dopamine system, particularly the VTA-NAc circuit, constitutes one of the major neurochemical pathway for reward (Everitt and Robbins, 2005) and alcohol is a well-known elicitor of extracellular dopamine at the NAc (Koob, 2013). During the adolescence, the top–down control of prefrontal cortex over the VTA-NAc circuit is relatively weak, resulting in a chronically VTA-induced activation of NAc (Crews et al., 2007). The latter could be potentiated by synaptic changes evoked by ethanol, especially those originated as cause of impaired gliotransmission. In this context, we believe that uncontrolled opening of hemichannels and pannexons in activated glial cells may enhance the dopaminergic drive of VTA on neurons of the NAc. Indeed, rodents chronically treated with ethanol exhibit increased levels of GFAP (Ortiz et al., 1995) in the VTA, while similar findings have been observed for different microglial inflammatory markers (He and Crews, 2008; Yang et al., 2014). At one end, astroglial hemichannel/pannexon opening potentiated by inflammatory mediators released from microglia, could promote presynaptic glutamate release in the VTA (**Figure 3**). Supporting this idea, high concentrations of glutamate at the synaptic cleft could be neurotoxic under pathological conditions (Lau and Tymianski, 2010; Ashpole et al., 2013). Importantly, glutamate released through glial hemichannels and pannexons triggers neuronal dysfunction and cell death as result of *N*-methyl-D-aspartate receptor (NMDAR) activation (Takeuchi et al., 2006; Orellana et al., 2011a,b). Presynaptic glutamate release may also be enhanced by the interaction of NMDARs and Panx1 channels in neurons. In fact, most of the glutamate released from glial hemichannels/pannexons modulate neuronal function by activating Panx1 channels in them (Orellana et al., 2011a,b). How NMDARs do elicit the activity of neuronal pannexons? A possible mechanism involves the phosphorylation of the C-terminal of Panx1 caused by the interaction of NMDARs with Src family kinases (Weilinger et al., 2016). It is possible that glial-induced changes in synaptic transmission at the VTA may relies on intracellular Ca^2+^ regulation depending on the opening of Panx1 channels in presynaptic and postsynaptic structures (**Figure 3**).

On the other hand, glial cells could increase VTA dopaminergic drive by decreasing inhibitory synapses into VTA. Studies by Molander et al. (2005) revealed that GABAergic neurons at the NAc suppres in a tonic fashion the dopaminergic firing at the VTA via the activation of accumbal glycine receptors (Molander et al., 2005). In a follow-up study, they propose that astrocyte cell swelling evoked by acute ethanol treatment leads to an increase in extracellular taurine, a well-known agonist of glycine receptors (Adermark et al., 2011). An alternative mechanism by which astrocytes could be key players in the increased dopamine concentration in the NAc may reside in the release of taurine through hemichannels (**Figure 3**), as they have been reported to allow the release of this transmitter (Stridh et al., 2008). Up to now, the only attempt to evaluate the role of hemichannels in alcohol addiction corresponds to Bull and colleagues (Bull et al., 2014). They found that motivation to self-administer ethanol after 3 weeks abstinence was increased following microinjection of mefloquine and 18-a-glycyrrhetinic acid at the NAc, two unspecific and general blockers of gap junction channels, hemichannels and pannexons (D’hondt et al., 2009). Without doubt, the interpretation of these findings in terms of the VTA-NAc circuit is complex, as these blockers have the potential to act in different brain cell types, each of them expressing their own array of connexin and pannexin-based channels. The intracerebral injection in limbic regions of specific mimetic peptides that selectively distinguish hemichannels v/s gap junction channels and pannexons (e.g., Gap19, TAT-L2) will disentangle the contribution of connexins and pannexins in alcohol addiction.

## Melanocortin-Dependent Impairment of Glial Cells and its Consequences on Brain and Peripheral Function During Alcoholism

Episodes of adolescent binge drinking could have long-term consequences that will affect not only the circuits involved in alcohol reward, but also those implicated in memory, learning and feeding behavior. The latter may in addition influence and disturb whole body metabolism and energy balance. In the following sections, we discuss in brief how ethanol-induced impairment in MC system may influence synaptic plasticity and peripheral metabolism, in particular, skeletal muscle.

### Alcohol Abuse during Adolescence and Synaptic Communication: Possible Role of Melanocortin Networks

Accumulative evidence suggests that prolonged alcohol consumption affects memory and cognitive processes (Zeigler et al., 2005), which are well-established indicators of CNS integrity and function. For instance, hippocampal neurons chronically exposed to ethanol exhibit an increased glutamatergic drive, i.e., increased levels of extracellular glutamate and alterations in its receptors and transporters (Tsai and Coyle, 1998; Krystal et al., 2003). Clinical studies have shown that there is a direct correlation between alcohol dependence and levels of glutamate in the cerebrospinal fluid (CSF) (Umhau et al., 2010). An imbalance in glutamate levels can affect the dynamics of glutamate receptors, however, alcohol can also directly affect the activity of glutamate receptors. Specific alterations in NMDARs include perturbations in the direct occupancy of receptors, alterations in gating, as well as changes in the phosphorylation and activation states of NMDARs (Lovinger et al., 1989; Woodward, 2000). Moreover, alcohol can also influence α-amino-3-hydroxy-5-methyl-4-isoxazolepropionic acid receptors (AMPARs) by inducing an increase in their expression and localization (Chen et al., 1999; Christian et al., 2012). In the mammalian CNS, both AMPARs and NMDARs mainly mediate fast excitatory neurotransmission, and participate directly in the control of synaptic transmission and plasticity (Traynelis et al., 2010).

The Rosetta stone of synaptic plasticity is LTP. This phenomenon entails a long-lasting enhancement of synaptic transmission between two neurons after high frequency stimulation, which results in the strengthening of neuronal synapses (Bliss and Collingridge, 1993). Notably, ethanol exposure dramatically blunts the induction of LTP (Givens and McMahon, 1995; White et al., 2000) and induces loss of hippocampal-dependent memory (Melia et al., 1996). How do MCs participate in the ethanol-induced alterations in synaptic plasticity? Several extracellular factors, including MC peptides such as α-MSH and their receptors (e.g., MC4R), modulate hippocampal synaptic transmission (Shen et al., 2013). In fact, d-Tyr-MTII, an agonist of MC4Rs, increases LTP in hippocampal slices by a mechanism involving the PKA-dependent insertion of AMPARs into presynaptic sites (Shen et al., 2013). PKA is an important target of MC4Rs and is a key player in plasticity-related events (Abel and Nguyen, 2008). Alcohol-mediated alterations in NMDARs and their function in synaptic plasticity is likely to have a structural basis. Chronic administration of ethanol decreases neuronal spine density, in particular that in mature cells (Korkotian et al., 2015). Importantly, downregulation of MC4Rs dramatically abolishes the increase in mature spines triggered by d-Tyr MTII (Shen et al., 2013), suggesting the possibility that ethanol-induced structural synaptic changes may involve impairments in MC4Rs. Further studies are needed to understand the underlying basis by which ethanol influences synaptic transmission, and whether glial cells play a role in this process.

### Heavy Drinking in Adolescents and Melanocortin-dependent Control of Whole Body Metabolism: Focus on Skeletal Muscle

Skeletal muscle is a dynamic and highly plastic tissue that adapts to various external stimuli (e.g., contractile activity, loading conditions and substrate supply) to match structural, functional, and metabolic demands (Bassel-Duby and Olson, 2006). This tissue plays a critical role in glycemic control and metabolic homeostasis, and is the predominant site of glucose disposal under insulin-stimulated conditions (DeFronzo et al., 1981). In this context, exercise increases skeletal muscle glucose uptake via an insulin-independent pathway (Lee et al., 1995), indicating that muscle contraction directly impacts on glucose homeostasis, and increases insulin sensitivity and glucose uptake in skeletal muscle fibers (Pereira and Lancha, 2004; Holloszy, 2005; Rose and Richter, 2005). Glucose uptake is important for the actions of both exercise and insulin at skeletal muscle fibers (Sakamoto et al., 2002; Pereira and Lancha, 2004). Each stimulus results in the redistribution of the glucose transporter type 4 (GLUT4) from intracellular vesicles to the sarcolemma, increasing the rate of glucose uptake into muscle fibers (Douen et al., 1990; McCarthy and Elmendorf, 2007; Foley et al., 2011).

Alcohol has profound effects on muscle and whole-body fuel metabolism, thus contributing to increased morbidity and mortality in people with alcohol dependence (Steiner et al., 2015). In fact, alcohol abuse increases the synthesis and secretion of various catabolic agents, such as inflammatory cytokines and glucocorticoids, as well as the production of oxidative metabolites generated by the hepatic metabolism of ethanol (Avogaro and Tiengo, 1993; Gonzalez-Reimers et al., 2011). Interestingly, Molina and colleagues found differential glucose uptake in muscles from rats exposed to alcohol (Molina et al., 1991), whereas studies carried out in healthy people show that alcohol acutely decreases insulin-stimulated whole-body glucose uptake (Yki-Jarvinen and Nikkila, 1985; Avogaro et al., 1987; Avogaro et al., 1996). While there is no consensus about the mechanism underlying alcohol-induced insulin resistance, it appears that alcohol may alter the actions of insulin at a number of key regulatory steps, including PI3K/Akt signaling pathways and/or GLUT4 translocation (Wasserman, 2009). For instance, alcohol intake reduces GLUT4 protein in the plasma membrane fraction of the gastrocnemius, but not in whole muscle homogenate from rats (Wilkes and Nagy, 1996; Lang et al., 2014). Similarly, *in vitro* incubation of myotubes with alcohol acutely inhibits insulin-stimulated GLUT4 translocation (Yu et al., 2000).

The brain regulates most energy metabolism in muscle (Braun and Marks, 2011), however, whether alcohol-induced impairment in muscle energetics and peripheral metabolism occurs as result of alterations in central neuronal circuits has not yet been examined. Energy homeostasis, the balance between caloric intake and energy expenditure, is regulated by the neuroendocrine and autonomic systems which are both controlled by the CNS. Specific neuronal circuits located in the hypothalamus and brain-stem continuously monitor signals reflecting energy status, and initiate appropriate behavioral and metabolic responses to deal with nutrient availability (Seeley and Woods, 2003; Lam et al., 2005; Plum et al., 2006). One of these networks is the MC system, which governs and modulates nutrient intake and energy metabolism (Williams et al., 2011; Edenberg and Foroud, 2013). MC3Rs and MC4Rs are the most relevant receptors involved in the regulation of energy homeostasis in different tissues (Fan et al., 1997), including skeletal muscle (Gavini et al., 2016). Recently, Gavini et al. (2016) found that the activation of MC receptors in the VMH increased heat dissipation in the gastrocnemius muscle during controlled activity, as well as augmenting skeletal muscle norepinephrine turnover and the expression of mediators of muscle energy. Furthermore, MC receptors play a critical role in appetite control and body-weight regulation, and are involved in obesity and diabetes mellitus type 2 (DM2; Cone, 1999; Nogueiras et al., 2007). In rodent models, activation of MC4Rs (Nargund et al., 2006) or ablation of AgRP/NPY-coexpressing neurons (Bewick et al., 2005; Gropp et al., 2005; Luquet et al., 2005; Ste Marie et al., 2005) results in anorexia and weight loss, whereas downregulation of MC3Rs, or the removal of agonist-producing neurons, leads to hyperphagia and obesity (Huszar et al., 1997; Chen et al., 2000). The role of MC3Rs in energy homeostasis is unclear, however, they may be one of the major receptors responsible for the anti-inflammatory properties of MC peptides, and perhaps owing to this role may influence energy homeostasis (Mountjoy, 2010a,b). Of relevance to this point is the fact that both obesity and DM2 are associated with chronic low-grade inflammation, caused by an imbalance between pro- and anti-inflammatory cytokines.

Complementary studies in patients (Festa et al., 2000; Calder et al., 2011; Donath and Shoelson, 2011) showed that blockade of central MC4R signaling promotes insulin resistance in skeletal muscle (Mountjoy, 2010b). Furthermore, loss-of-function mutations in MC4Rs are associated with hyperphagia, severe early onset obesity, hyperinsulinemia, and increased lean mass (Vaisse et al., 1998, 2000; Yeo et al., 1998; Farooqi et al., 2003; Yeo et al., 2003; Biebermann et al., 2006). Similar effects have also been observed in studies with MC4R knockout mice (Huszar et al., 1997). These findings support an important role for the MC system in whole-body and muscle energy homeostasis across mammalian species. Thus, MCs and their receptors could be one of the major ways by which ethanol exerts its effects on metabolism and muscle in people with alcohol dependence. In fact, neuronal networks that sustain MC signaling are major targets for crucial endocrine messengers and hormones that control energy demands and body weight, including leptin, insulin, cholecystokinin, and ghrelin (Moran, 2004; Cone, 2005; Aja and Moran, 2006; Harrold and Williams, 2006; Moran, 2006; Xu and Barsh, 2006). Indeed, the crucial mechanisms by which insulin and leptin govern energy homeostasis are determined by their influence on hypothalamic POMC or AgRP neurons (Varela and Horvath, 2012). For example, the actions of insulin in AgRP neurons reduces gluconeogenesis in the liver, but in POMC neurons has an inverse effect, favoring energy expenditure in an MC-dependent manner (Lin et al., 2010). In the same manner, the MC system directly controls hepatic glucose metabolism (Obici et al., 2001), and thermogenesis in brown adipose tissue (Voss-Andreae et al., 2007) and skeletal muscle (Gavini et al., 2016), revealing the existence of direct neuroendocrine control of MCs over peripheral cell metabolism.

## Conclusion and Future Directions

Decreased α-MSH drive, glial inflammation, increased hemichannel, and pannexon opening, and over activation of VTA-Nac circuit may constitute an interdependent cyclic process during heavy alcohol drinking. The latter could be potentiated during adolescence and thereby, whether interruption of any of these steps can ameliorate the cascade of events that lead to alcohol addiction could be crucial to interrupt further chronic alcoholism in adults. Because ethanol-induced decrease in α-MSH drive may potentiates glial inflammation in other brain areas including the hippocampus and hypothalamus, its impact on synaptic transmission and memory, as well as whole body metabolism and energy expenditure could be critical to ameliorate the major devastating effect of heavy drinking. Accordingly, the different components of MC system may serve as potential targets for therapeutic interventions in alcohol abuse among adolescents and later in the adulthood. Nevertheless, further studies are required to determine how gliotransmission mediated by hemichannels and pannexons contributes to ethanol addiction and drinking behaviors.

## Author Contributions

JAO: Conceived Idea, wrote paper and some illustrations; WC: Conceived idea, wrote paper; MC: Conceived idea and wrote the paper; JL: Conceived the idea and wrote some parts of the paper; EK: wrote the paper; CO-F: Conceived idea and wrote some parts of the paper; RQ: Conceived the idea, wrote the paper and revised final version.

## Conflict of Interest Statement

The authors declare that the research was conducted in the absence of any commercial or financial relationships that could be construed as a potential conflict of interest.
